# External Cervical Resorption Detected by Cone Beam Computed Tomography on an Immature, Unerupted Maxillary Second Premolar

**DOI:** 10.1155/2024/6590778

**Published:** 2024-06-06

**Authors:** Karen Berrigan, Bradly Gettleman, Sabita Rao, Janet Jordan, Mark Stieg, Lawrence Johns

**Affiliations:** Faculty of Midwestern University Dental Institute, Glendale, Arizona, USA

## Abstract

Root resorption is a commonly recognized risk of orthodontic treatment and can range from minimal changes at the apex to aggressive and extensive erosion of tooth structure. The following report is aimed at presenting a rare case of idiopathic aggressive preeruptive root resorption in a maxillary second premolar of a young child prior to orthodontic force. During phase I orthodontic treatment, the maxillary premolar required surgical exposure with a bracket and chain to assist in its eruption. Before the exposure, a cone beam computed tomography (CBCT) was captured to aid in localizing the premolar and its relationship to vital structures. In addition to identifying relevant anatomy, the image also revealed an incidental finding of extensive external cervical root resorption. The risks, benefits, prognosis, and alternatives of continuing with surgical exposure of the affected premolar were presented to the guardian. The informed and consented decision was made to continue with the exposure and subsequent orthodontic treatment to align the tooth in the arch. Upon eruption to the occlusal plane, the tooth was amenable to endodontic pulp vitality testing, Endo Ice, and a follow-up CBCT. It was determined that there was advanced aggressive progression of the resorptive process. The Patel Classification 3Cd/Class 4 Heithersay ECR diagnosis and a poor prognosis ultimately resulted in the extraction of the tooth. Management of this case highlights a number of important clinical features including the rarity of advanced preeruptive idiopathic external cervical root resorption, a multidisciplinary approach (orthodontic, endodontic, and surgical) to diagnose and manage the ECR, and the importance of prescribing and acquisition of appropriate imaging to aid in the early diagnosis of the entity. This case report will add to the body of knowledge of a rare incidence of advanced ECR on a young patient with a nontraumatized, unerupted maxillary second premolar.

## 1. Introduction

A common risk of orthodontic treatment is the potential for root damage resulting from external resorption [1]. Orthodontic external root resorption may have an apparent etiology, or it may be idiopathic. The loss of the protective root cementum surface may predispose the root to irreversible damage or, in severe cases, tooth loss. External cervical root resorption (ECR), an idiopathic type of destructive root resorption, is often difficult to detect with conventional two-dimensional imaging techniques.

Orthodontic diagnostic workups typically include a panoramic radiograph for screening and early detection of anomalies, potential pathologies, incidental findings, and valuable information about tooth position, root lengths, and shape [2, 3]. To assist in predictable treatment outcomes, suspicious findings may prompt further radiographic imaging [4]. Appreciation of root length, shape, and width can influence orthodontic treatment options, treatment length, and mechanics of tooth movement. Early modes of detailed diagnostic imaging relied on intraoral periapical radiographs. Adequate clarity of all anatomical surfaces essential for proper diagnosis is not always evident on periapical radiographs [5, 6]. One such structure is the circumference of root surfaces which is best imaged with cone beam computed tomography (CBCT) [6]. Recent studies of CBCTs document a high incidence of ECR incidental findings that are otherwise undetected on routine radiographic imaging [7]. A conclusion might be drawn that without the benefit of CBCT, ECR is underdiagnosed [6, 7].

Many factors are considered in establishing orthodontic treatment goals and options. Patient chief complaints in addition to routine orthodontic diagnostic records are essential to comprehensive treatment planning [8]. The following novel case report sequentially highlights a first phase of orthodontic treatment to recapture lost arch length, in addition to maintaining space for future eruption of a contralateral maxillary second premolar. Failure of the contralateral, nonimpacted second premolar to naturally erupt into the arch lead to the eventual surgical exposure with bracket and chain for orthodontic traction. Use of CBCT to assist the surgeon in anatomical orientation and identification of structures lead to the incidental discovery of advanced ECR of the nonimpacted, unerupted, immature second premolar with no history of trauma.

CBCT is an essential diagnostic tool when surgical exposure of unerupted teeth becomes necessary [5]. In this case, the CBCT's role was fundamental in identifying an aggressive ECR in the unerupted immature maxillary second premolar.

## 2. Case Report

A nine-year and 7-month-old male patient presented with his guardian, requesting a second opinion for orthodontic treatment.

The patient's height and weight were age appropriate, with unremarkable medical history, and the patient appeared to be socially well adjusted. A detailed family history was recorded with no previous reports of trauma, impacted teeth, or family members having orthodontic treatment.

**Figure 1:**
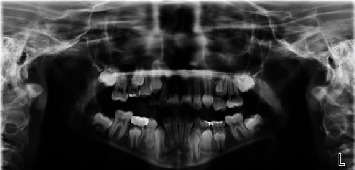
Initial screening panoramic radiograph.

Clinical examination revealed a mixed dentition malocclusion with slight excess overjet, deep overbite, moderate arch length discrepancies, and a palatally blocked out maxillary left second premolar. A screening panoramic radiograph (Planmeca Promax 6114 Helsinki, Finland) (Figure 1) was captured to evaluate the dentition and to determine if further diagnostic images are necessary. The image revealed that all permanent dentition was present and at an age appropriate stage of development. The maxillary left second premolar was palatally blocked out due to the premature loss of deciduous second molar J and mesial drifting of #14 with subsequent loss of arch length. Deciduous tooth #A presented with two minor occlusal restorations and minimal evidence of remaining root structure. Of particular interest was the lack of eruption to the occlusal plane of primary tooth #A, in addition to the apically positioned succedaneous tooth #4. Primary tooth #T presented with a stainless steel crown, without pulpotomy. A lateral cephalometric radiograph reveals a Class I skeletal pattern with good growth potential. There was ideal symmetry, smile line, and profile.

A first phase of orthodontic treatment was proposed to recapture adequate arch length for #13 as well as space maintenance for future eruption of #4. Orthodontic records were reviewed with the guardian and patient. Treatment options were proposed including the option of extraction of #4 and #13 with the possibility of incomplete space closure. The guardian and patient opted for recovering space for #13 and maintaining arch length for #4.

Prior to starting orthodontic treatment, all routine orthodontic risks, including root resorption, bone loss, tooth loss, and the need for future phase II orthodontic treatment, were described. Discussions also included the possible need for surgical exposure with bracket and chain of unerupted teeth. Informed consent was read and signed by the guardian of the patient.

**Figure 2:**
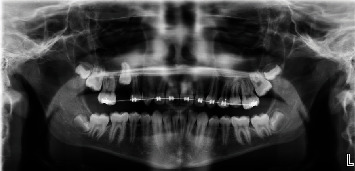
Progress panoramic radiograph eleven months into orthodontic treatment, # A still retained.

**Figure 3:**
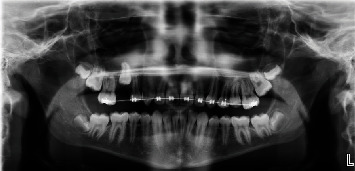
Progress panoramic radiograph, twelve months after extraction of #A.

**Figure 4:**
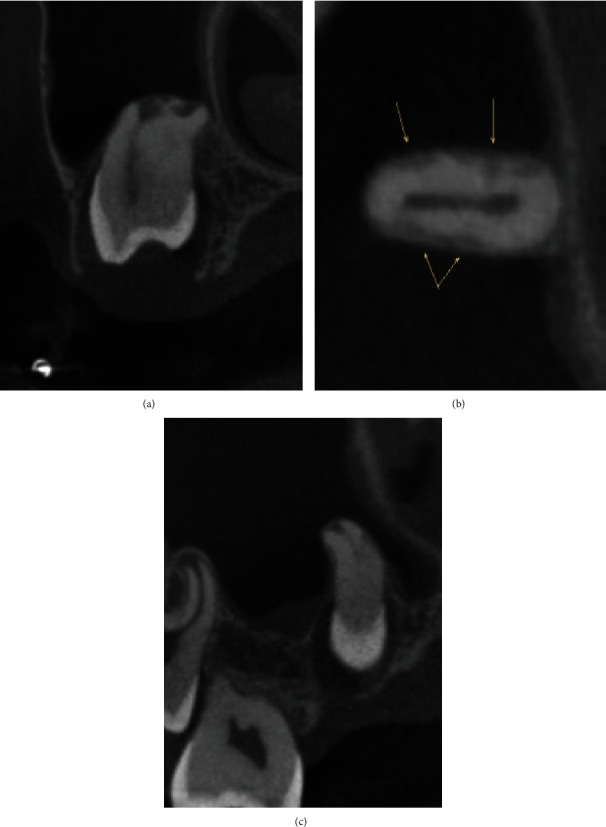
Initial CBCT: (a) coronal section, advanced ECR at apex, (b) axial section arrows indicating lateral extensions, and (c) sagittal section showing lateral extensions.

**Figure 5:**
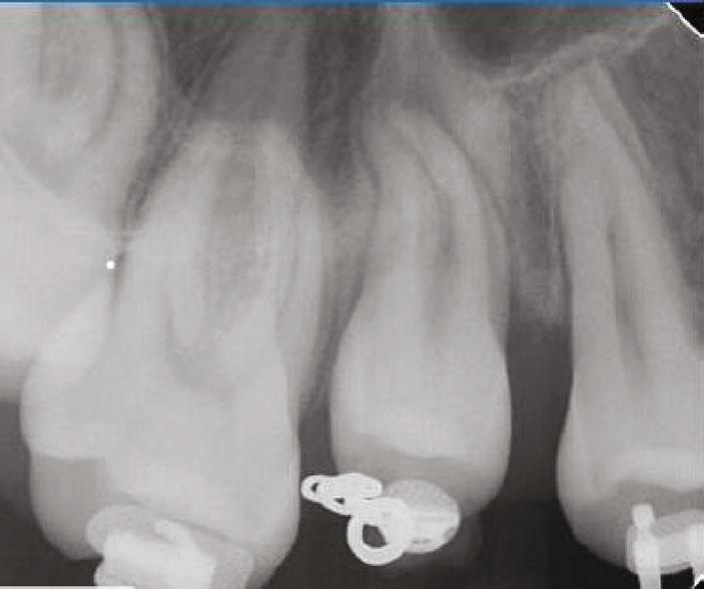
Enlarged follow-up periapical radiograph tooth #4, 6 months postsurgical exposure.

**Figure 6:**
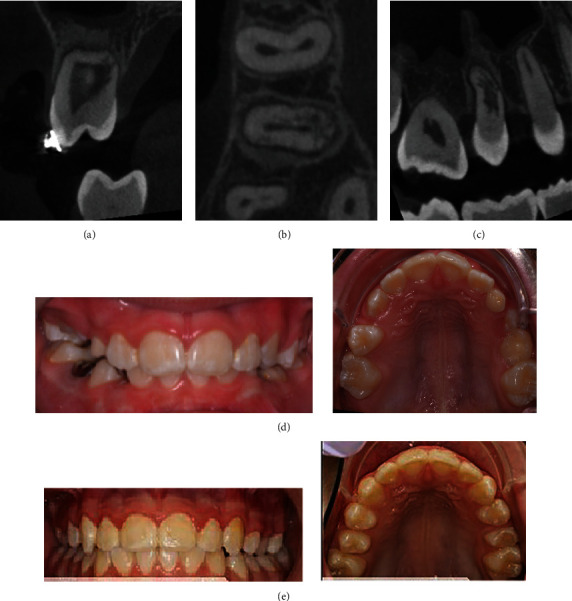
Follow-up CBCT eight months after surgical exposure. Tooth #4 eruption near to occlusal plane: (a) coronal, (b) axial, and (c) sagittal. Significant progression of resorption. (d) Initial intraoral images and (e) final intraoral image.

Maxillary orthodontic appliances (Unitek Victory series, slot size 0.022 × 0.028 bonded with Unitek Transbond XT) were placed without incident, and attention was initially directed to regaining space for #13. A final leveling arch wire 0.018 × 0.025 NiTi was used as space was eventually created for #13. After repeated failure by the patient to exfoliate tooth #A, a progress panoramic radiograph (Figure 2) was captured. At that time, it was deemed necessary to extract #A as soon as possible. Atraumatic removal of primary tooth #A, at fourteen months of treatment, allowed adequate space for eruption of #4. After an additional twelve months of failure to naturally erupt, a second progress panoramic radiograph (Figure 3) was captured to determine the etiology. The panoramic radiograph revealed possible root dilaceration of #4. Root dilacerations can complicate eruption of permanent teeth. This finding was reviewed with the guardian and patient. Treatment options were discussed including extraction with possible incomplete space closure or surgical exposure with bracket and chain to assist in orthodontic eruption. The guardian and patient were not interested in extraction, and the decision was made to pursue surgical exposure with bracket and chain. At twenty-seven months of treatment, a CBCT scan (Figure 4) (J Morita 3D Accuitomo system at 90.0 kVp, 8.0 mA, 17.5 seconds, resolution 0.08 mm, and field of view 40.2 mm × 40.2 mm × 24.8 mm) was captured for ideal surgical orientation and identification of anatomical structures. The CBCT analysis report, by a board-certified Oral and Maxillofacial Radiologist, describes unerupted #4 with “the root half formed and curves slightly to the distal; an irregularly shaped radiolucency was also noted at the mesiopalatal aspect, highly suggestive of resorption.” An endodontic consultation by a board-certified Endodontist determined that the radiolucency was most likely external cervical resorption (ECR). These findings were reviewed with the guardian and patient. The risk of further root resorption and the possible need for endodontic treatment in addition to potential loss of the tooth were discussed. In its current unerupted apical position, no endodontic treatment could be attempted. The guardian made the decision to proceed with surgical exposure with bracket and chain to assist in bringing #4 into the arch. Eruption of #4, near the occlusal plane, was uneventful. At six months postsurgery, a periapical radiograph (Figure 5) (Progeny 60kv, 7 mA, 0.125 s) revealed evidence of ECR progression. At eight months postsurgery, a follow-up CBCT (same settings as previous CBCT) (Figure 6) and endodontic consultation were repeated. The CBCT revealed continued aggressive progression of ECR on #4. Since the tooth was finally accessible intraorally, pulp vitality testing was performed using electric pulp test (EPT) and Endo Ice. The tooth responded at 46/80 on the EPT and positive short duration to Endo Ice indicating no inflammatory changes within the pulp. Progression of ECR is evident in the progress CBCT (Figure 6). Treatment options including endodontic therapy and repairing the resorptive defect were discussed. Due to the advanced resorption, prognosis with endodontic treatment was deemed poor, and the decision was made to extract the tooth. At thirty-seven months of treatment and in consideration of poor oral hygiene, extensive appliance breakage, and the final treatment decision to extract #4, the orthodontic appliances were immediately removed. A Hawley retainer was placed to maintain alignment and hold the existing arch length. Uneventful extraction of the second premolar and granulation tissue was completed after appliance removal. The guardian was made aware that all facial growth must be completed prior to implant replacement of #4. The patient presented for only two retainer follow-up appointments at four and 8 weeks postorthodontic treatment. Compliance was poor with his retainer, and loss of arch length was noted. The patient is no longer available for continued monitoring.

## 3. Discussion

Root resorption may be categorized as either physiologic or pathologic resulting in a loss of cementum, dentin, and/or bone [9]. Physiologic resorption of the primary dentition is a naturally occurring process that is necessary for the eruption of succedaneous teeth [10, 11]. Root resorption that occurs in permanent teeth is a pathologic process that may result in irreversible damage and eventual loss of dentition [12]. Pathologic root resorption can be further classified as internal or external depending on the location of its origin [13]. Internal resorption has its origins within the pulpal sac/chamber and destroys the root canal walls and possible invasion to the cementum [9, 14]. In contrast, ECR is initiated on the cementum surface of the root and is classified depending on its point of origin [9].

Known common resorptive lesions include surface resorption, external inflammatory resorption, external replacement resorption, transient apical breakdown, and external cervical resorption [13]. Our report of ECR is a distinct entity that has been classified and described by Heithersay [15] and Patel et al. [16]. ECR has the potential to be invasive and destructive in nature, and its progression is considered complex [17]. The exact etiology remains largely unknown and may be due in part to an anatomical variation in the cementoenamel junction (CEJ) [18]. Microscopic analysis of the CEJ in teeth exhibiting ECR reveals scattered gaps in the protective cementum layer. Although minor and scattered, the lack of protective cementum increases the potential for exposure of the underlying dentin. This exposed dentin is vulnerable to osteoclastic resorption and results in breakdown of the root surface [12, 19]. The ensuing inflammation creates a resorptive defect which then progresses apicocoronally and circumferentially eventually directing to the pulp chamber [12, 18].

Others argue that the etiology in the initial stages of ECR is the result of disruption of the PDL. This disruption leads to the formation of a blood clot and subsequent focal inflammation. Granulation tissue formation from the adjacent tissues may reach the exposed dentin which is now vulnerable to resorption. Continued progression of ECR is dependent on bacterial invasion or mechanical forces [17]. Our case differs in presentation of typical ECR also referred to as external invasive resorption (EIR) [20] due to the tooth's superior position within the alveolus, no known mechanical forces, history of trauma, or associated risk factors [12, 21].

A common ECR classification system identifies four stages of degradation spanning the spectrum from small cementum defects (class I) to near complete cavitation of the root (class IV) [21]. Increased specificity and sensitivity of CBCT have established an enhanced classification system to assist in diagnosis and treatment outcomes [16].  Figure 4, captured prior to surgical exposure, clearly shows immature development of the root length, lack of communication of the tooth to the oral environment, and also the extent of root destruction both laterally and apically.  Figure 4(c) shows that the root resorption is in the middle to apical one third of the root, circumferentially extends 180 degrees to approximately 270 degrees, yet is confined to the dentin which defines this lesion as 3Cd [16].

ECR lesions are typically painless until there is invasion into the pulp chamber [12]. Since the eroded surface is not always visible on routine radiographs, these lesions often progress to later stages before they are recognized [18].

The case we present shows initial evidence of advanced ECR in an unerupted, nonimpacted tooth with immature root development and no history of trauma. There are numerous case reports in the literature of preeruptive intracoronal resorption [22, 23]; however, there is little published evidence of preeruptive ECR that is not accompanied by trauma or impactions [15, 24]. Recent publications document evidence of invasive resorption due to surface defects in unerupted teeth [20]. Our initial CBCT clearly shows external communications of the root surface with destruction evident in both a longitudinal pattern and also erosive lateral extensions in the more apical area of the root (see Figures 4(a), 4(b), and 4(c)). Although we estimate that one half of the root development was completed at the initial CBCT, there is no evidence of erosion at the CEJ. While ECR is most frequently seen at the CEJ, this is not the sole diagnostic criteria [12, 25]. The use of CBCT has simplified and increased the opportunity to identify this aggressive destructive lesion at an earlier stage of development [18]. Early detection allows the practitioner to advise the patient of the location and severity of the lesion, thus allowing for appropriate treatment options. Our initial screening panoramic radiograph (Figure 1) did not indicate any sign of erosive root loss. Only after the decision was made to surgically expose #4 and orthodontically erupt the tooth into the arch, the decision to acquire a CBCT was made as well (Figures 4(a), 4(b), and 4(c)). Accurate diagnosis and review of the CBCT with the guardian and patient allowed the opportunity to set a realistic expectation and prognosis for this tooth. Evidence supports the theory that reparative mineralized tissue, similar to bone, substitutes the resorbed tissue when the dentin is in direct contact with the bone [17]. This boney substitution can result in the fusion of the alveolar bone with the dentin, similar to ankylosis. With this in mind, it was deemed an acceptable treatment to surgically expose the tooth, place a bracket and chain, and erupt the tooth into the arch as much as possible. If the tooth was maintained in its original apical position, endodontic treatment would be impossible and could result in progressive root destruction with the likelihood of ankylosis. Removal of an ankylosed tooth, with its close proximity to the maxillary sinus and related structures, poses an increased risk of damage to anatomical structures. Also to be considered is the difficulty of treating advanced ECR and the high probability of failure [12]. After eruption into the oral environment, a follow-up CBCT was captured (Figure 6) showing progression of ECR to classification 4Dp [16]. Prognosis of advanced ECR is poor, and the guardian consented to removal of #4.

Known predisposing factors associated with ECR include intracoronal bleaching, trauma, orthodontics, any orthognathic or dentoalveolar surgery, periodontal treatment, bruxism, intracoronal restorations, developmental defects, and systemic diseases [12, 15, 26, 27]. Our case has no history of direct orthodontic force, trauma, bleaching, dentoalveolar surgeries, periodontal treatment, bruxism, malocclusion, intracoronal restoration of the affected tooth, and developmental or systemic issues. Recent reports of undiagnosed ECR in an impacted mandibular second premolar raise the possibility that other cases may also present with ECR prior to orthodontic treatment [26]. Our case differs in location, unerupted maxillary second premolar, and mixed dentition at time of treatment and ECR diagnosis.

Orthodontically induced inflammatory apical root resorption is a known risk of orthodontic treatment [1, 28, 29] and is one of the many known resorptive processes [25]. Studies have documented frequency and degree of orthodontically induced apical root resorption [28]. Most studies agree that orthodontic apical root resorption is commonly seen in the anterior teeth especially maxillary lateral incisors followed by maxillary and mandibular second premolars [1, 30]. In the case we present, no direct orthodontic forces were applied prior to the discovery of ECR, and we conclude that this is not orthodontically induced apical root resorption.

A recent study using CBCT assessment documents that of the 168 incidence of ECR, only 9.5% was detected in maxillary premolars [18]. Our case report may be unique in presentation due to the advanced resorption, immature status of the tooth, and its position at initial diagnosis.

The patient's primary teeth were not subject to any known traumas, bruxism, or direct orthodontic forces. Although tooth movement in the proximity of primary tooth #A and #4 may have contributed minor force to adjacent bone, no direct tooth movement was realized on these teeth.

## 4. Conclusion

The aim of this case report is to provide evidence of advanced external cervical resorption in an unerupted tooth of a young patient. Clinicians should be aware that the incidence of ECR may be underreported especially in the pediatric population. Suspicion of invasive root resorption in unerupted teeth should not be ruled out even if the root has not completed development and does not fit the known ECR etiologies. Delayed development and eruption of natural dentition should prompt a practitioner to request advanced imaging for thorough diagnosis, prognosis, and treatment planning.

Although cases of advanced cases of ECR are typically seen in older patients, our case illustrates the potential for damage at a very early stage of development.

## Data Availability

Data sharing is not applicable to this article as no new data were created or analyzed in this study.
